# LATENT VARIABLE MODELING FOR BIOLOGICALLY INFORMED PROGNOSIS IN STUDIES OF PHYSICAL RESILIENCE

**DOI:** 10.1093/geroni/igad104.1824

**Published:** 2023-12-21

**Authors:** Karen Bandeen-Roche, Qianli Xue, Ravi Varadhan

**Affiliations:** Johns Hopkins University, Baltimore, Maryland, United States; Johns Hopkins, Baltimore, Maryland, United States; Johns Hopkins University School of Medicine, Baltimore, Maryland, United States

## Abstract

Physical resilience – rebound in relevant functioning and biomarkers following a health stressor – is hypothesized to be rooted in the level of fitness of stress-response physiology defining one’s “physiological resilience capacity” (PRC). This physiology forms a dynamical system comprising specific modules (the individual stress response systems and their underlying components) and their dynamic interactions with each other via feedback and other protocols. Such a system can be modeled using differential equations whose parameters may then define the PRC. We do not yet know how to measure these parameters directly, however. Rather, they are conceptually defined “constructs” which must be inferred using indirect measures—ideally, stimulus response data probing multiple aspects of the relevant physiology. Latent variable models are ideally suited to this setting. Two challenges for their application in studies of resilience are presented: (1) Integrating specific scientific knowledge on the dynamical systems in modeling the co-distribution of the indirect measures. (2) Synergizing such models’ advantages for construct measurement with advantages of machine learning approaches to optimize accuracy for predicting resilient outcomes by inferred PRC. Challenges and their proposed solutions are illustrated using multifaceted stimulus response data being collected in an ongoing investigation on the physiological basis of resilience to clinical stressors in older adults, the Study of Physical Resilience in agING. The work aims to produce physiologically rooted measures providing effective risk predictors for older adults facing impending stressors as well as intervention candidates by which to promote PRC in the longer term.

